# DDE, a Degradation Product of DDT, and Duration of Lactation in a Highly Exposed Area of Mexico

**DOI:** 10.1289/ehp.10550

**Published:** 2007-11-22

**Authors:** Lea A. Cupul-Uicab, Beth C. Gladen, Mauricio Hernández-Ávila, Jean-Philippe Weber, Matthew P. Longnecker

**Affiliations:** 1 Center for Population Health Research, Instituto Nacional de Salud Pública, Cuernavaca, Morelos, México; 2 Epidemiology Branch and; 3 Biostatistics Branch, National Institute of Environmental Health Sciences, National Institutes of Health, Department of Health and Human Services, Research Triangle Park, North Carolina, USA; 4 Subsecretaría de Promoción y Prevención de la Salud. Ministry of Health, México City, Mexico; 5 Centre de Toxicology, Institut National de Santé Publique du Québec, Québec, QC, Canada

**Keywords:** breast-feeding, DDE, DDT, infant, lactation

## Abstract

**Background:**

Higher levels of 1,1-dichloro-2,2-bis(*p*-chlorophenyl)ethylene (DDE), the major degradation product of 1,1,1-trichloro-2,2-bis(4-chlorophenyl)ethane (DDT), have been related to shorter duration of breast-feeding in previous studies. If DDE truly shortens lactation, this has public health importance regarding infant mortality and the use of DDT for malaria control.

**Objective:**

Our aim was to assess the relationship of maternal DDE concentrations with length of subsequent lactation.

**Methods:**

We conducted a relatively large study in a highly exposed area of Mexico. We followed 784 mother–son pairs to determine length of lactation. DDE and DDT were measured in maternal serum obtained within a day of delivery. We fit proportional hazard models with and without stratifying by previous breast-feeding, because an association of DDE with duration of lactation among those who breast-fed previously could be attributed to a noncausal mechanism.

**Results:**

Compared with those with DDE concentrations ≤ 3.00 μg/g, the adjusted hazard ratios of weaning according to DDE category were, for concentrations 3.01–6.00 μg/g, 1.27 [95% confidence interval (CI), 1.04–1.55]; for concentrations 6.01–9.00 μg/g, 1.23 (95% CI, 0.92–1.63); and for concentrations > 9.00 μg/g, 1.17 (95% CI, 0.92–1.49). The corresponding ratios for women who previously breast-fed were 1.40 (95% CI, 1.06–1.87); 1.91 (95% CI, 1.24–2.93); and 1.76 (95% CI, 1.22–2.53). Those for women who had not breast-fed previously were 1.14 (95% CI, 0.86–1.52); 0.90 (95% CI, 0.61–1.31); and 0.91 (95% CI, 0.66–1.26).

**Conclusions:**

Data from our relatively large study in a highly exposed area of Mexico did not support the hypothesis that exposure to DDE shortens length of lactation. The association seen in women who previously breast-fed was likely attributed to a noncausal mechanism. Nonetheless, whether DDT has other important adverse effects on humans is still an open question.

Mothers with higher levels of 1,1-dichloro-2,2-bis(*p*-chlorophenyl)ethylene (DDE), the major degradation product of 1,1,1-trichloro-2,2-bis(4-chlorophenyl)ethane (DDT), stopped breast-feeding their children sooner than those with lower levels of exposure in previous studies (Gladen and Rogan 1995; Karmaus et al. 2005; Rogan et al. 1987). Shorter duration of breast-feeding increases infant mortality (Gartner et al. 2005; Leon-Cava 2002); if DDT exposure truly shortens lactation, this has public health importance. DDT is still used for control of malaria in some countries [Curtis 2002; Longnecker 2005; World Health Organization (WHO) 2006], resulting in relatively high human exposure. Characterizing DDT’s human toxicity accurately is important for making good decisions about vector-control policy.

When the association of DDE with shortened duration of lactation was first reported, the possibility that a noncausal mechanism could account for the relation was recognized (Rogan et al. 1987). A woman who breast-fed longer in her first lactation will tend to breast-feed longer the second time as well, whereas a woman who breast-fed shorter once will tend to do so again. Also, because DDE is excreted in milk (Rogan et al. 1986), the woman with longer lactation would have eliminated more DDE via breast milk than the one who breast-fed less. That creates a noncausal association between higher DDE and shorter lactation in women who previously breast-fed. In the first study to examine the association, higher DDE was associated with shorter lactation even among those breast-feeding for the first time (Rogan et al. 1987), but a second study found an association only in women who previously breast-fed (Gladen and Rogan 1995). Subsequent reports have given inconsistent results (Karmaus et al. 2005; Weldon et al. 2006).

To evaluate the association with greater statistical power, we conducted a relatively large study in Tapachula, Chiapas, Mexico. This population was exposed for almost 40 years: DDT was used for agriculture until 1991 and for malaria control until 1998. The primary aim of the present study was to assess the relationship of maternal DDE concentrations and length of subsequent lactation in a cohort of 784 mother–son pairs in which maternal serum DDT and DDE levels at delivery had been previously determined.

## Materials and Methods

The participants were recruited from a previous cross-sectional study of newly-delivered male infants and their mothers (*n* = 872), conducted in 2002–2003 (Longnecker et al. 2007; Romano-Riquer et al. 2007). Because the objective of that study was to evaluate antiandrogenic effects of DDE, only boys were recruited. In the previous study, women were enrolled during the postpartum period at both of the city’s hospitals, which also serve the surrounding areas. Exclusion criteria for the mother included the following: age > 35 years; preeclampsia or pregnancy-related diabetes or hypertension; seizure disorders requiring daily medication; use of cortico-steroids; history of psychiatric, kidney, or cardiac disease or repeated urinary tract infections; and non-Spanish speaker. Infants were excluded if gestational age at delivery, as estimated by the Capurro scale (Capurro et al. 1978) or the medical record (based on last menstrual period), was < 36 weeks, birth weight was < 2,500 g, pregnancy was not singleton, Apgar score at 5 min was ≤ 6, or child required intensive care. The previous study included questionnaires, anthropometry, and collection of maternal serum for the measurement of DDT and DDE.

We conducted a follow-up study of these subjects to determine length of lactation. Mother–son pairs were eligible if they were enrolled in the cross-sectional study, the child was not given up for adoption (*n* = 1), and the mother had not used medications that inhibit or increase milk production, including contraceptive pills with estrogens, bromocriptine, or metoclopramide (*n* = 6). Eligible mothers were invited to participate and gave informed consent before participation in the study. The study protocol was reviewed and approved by the institutional review boards at the Instituto Nacional de Salud Pública in México and the National Institute of Environmental Health Sciences in the United States.

Of those who were eligible (*n* = 865), 90.6% (*n* = 784) were followed. Of the remaining 81 (9.4%), for 10 subjects the recorded address did not exist, for 59 the address was found but the mother was not, for 4 the mother or father refused to participate, and for 8 the mother or child had died.

Follow-up started on 20 January 2004. At that time, the ages of the babies were 3.3 to 25.1 months (median, 13.2). For children already weaned at the first visit, we recorded the duration of lactation. For those still being breast-fed, we continued home visits approximately every 2 months until the child was weaned.

The schedule of visits varied across the participants for logistical reasons. The median age at the first visit was 15.7 months (25th and 75th percentiles, 11.6 and 21.6 months). The number of visits ranged from 1 to 7 (median, 3), and the time between visits ranged from 0.8 to 11.4 months (median, 2.2). The interval from weaning until the next study visit (i.e., the recall period for duration of lactation) had a median of 7.4 months (quartiles, 2.4 and 14.1).

### Length of lactation

A questionnaire was administered to the mothers during each home visit. Interviewers received special training before beginning the study and periodic retraining throughout. Length of lactation was defined as the last time the child received any breast milk, regardless of the introduction of liquids or solid foods. Mothers were first asked whether they ever breast-fed the child and, if so, whether they were currently breast-feeding. If they had stopped, we asked the number of months they had breast-fed and the date they completely stopped. Twenty-six inconsistencies were corrected; if the mother said at one visit that she was still breast-feeding, but at the next visit said she weaned before the age at the previous visit, we used the age at the previous visit as the duration. For 11 mothers (1.4%) who reported that their attempt to breast-feed failed, a duration of 5 days was assigned. If the child was not weaned by the last study visit (*n* = 97, 12.4%), duration was censored at that time.

### DDE and DDT

DDE and DDT were measured in maternal serum obtained within 1 day of delivery. DDE and DDT were quantitated after solid phase extraction (C_18_ column purification), using gas chromatography with mass spectrometry detection (Saady and Poklis 1990; Smith 1991). The limit of detection for both analytes was 0.2 μg/L. The between-assay coefficients of variation were 7% for DDE (at 10 μg/L) and 6% for DDT (at 2.5 μg/L). A recovery of 97% was obtained for both analytes. Total serum lipid content was estimated based on levels of free and total serum cholesterol, triglycerides, and phospholipids (Patterson 1991), measured using standard enzymatic methods. DDE and DDT concentrations were expressed as micrograms per gram of lipids.

### Statistical analysis

To test the hypothesis under study, we used a Cox proportional hazard model for weaning. We used Kaplan-Meier survival curves to describe unadjusted duration. All analyses were done using Stata Statistical Software (release 9.0; StataCorp, College Station, TX, USA).

As described above, an artifactual association of DDE with shorter lactation duration can occur in women who previously breast-fed, but not in women who did not. A true association should be observed in both groups. Thus, we first constructed models using all participants, but then also stratified on previous breast-feeding.

In the primary analysis, DDE categories were defined as ≤ 3 μg/g, 3.01–6 μg/g, 6.01–9 μg/g, and > 9 μg/g. These categories were chosen before examining the DDE–outcome relationships, and resulted in a large exposure contrast between those in the high and low categories while maintaining adequate numbers for analysis. For DDT, we used these categories: ≤ 0.25 μg/g, 0.26–0.75 μg/g, 0.76–1.99 μg/g, and ≥ 2 μg/g.

Variables considered *a priori* as potential confounders were hospital of recruitment, rural/urban residence, mother’s age at birth, education (none, 1–6, 7–9, 10–12, ≥ 13 years), prepregnancy body mass index (BMI), smoking (ever, never), poverty status (poorest, somewhat poor, not poor) and previous breast-feeding (yes, no). We used weight at the first follow-up visit to calculate BMI if the prepregnancy weight was missing (*n* = 127, 16.2%). Smoking referred to any time before or during the pregnancy; only 10 women smoked during pregnancy.

We measured poverty status using national standards, based on monthly per capita income in Mexican pesos. In urban areas, those with an income < 672 Mexican pesos were in the poorest category; they would have difficulty buying adequate food. Of the remainder, those < 1,367 pesos lacked adequate income for other human needs, so were still considered poor. In rural areas, the cut points were 495 and 946 pesos, respectively [SEDESOL (Secretaría de Desarrollo Social) 2002]. If income at recruitment was missing (*n* = 77, 9.8%) we used income at the first follow-up visit.

Our model assumes proportionality of the hazards. Because residence area and hospital of recruitment were important confounders and were not proportional, we stratified the baseline hazard by these two variables.

We evaluated additional confounders using the change in estimate method, starting with all variables and deleting one by one in a stepwise way (Greenland 1989). A variable was considered a confounder if removing it caused a change in the hazard ratio for DDE (considered both as a continuous and categorical variable) of ≥ 10%. Potential confounders were mother’s alcohol use, whether the mother received support for breast-feeding, timely initiation of breast-feeding, trimester of first prenatal consultation, type of delivery, whether the father was living with the mother at the time of delivery, whether the mother received instructions about how to breast-feed, baby’s year of birth, and nipple anatomy problems such as flat or inverted nipples. Support for breast-feeding (e.g., orientation, motivation, and help with home tasks) was defined as any noneconomic help that the mother received from relatives or people close to her. Initiation was considered timely if the baby began suckling within 1 hr of birth (Setty 2006). None of these variables was shown to be a confounder in the whole population or in either stratum of previous breast-feeding.

We looked also for interactions of DDE (considered as a continuous variable) with poverty index, mother’s BMI before pregnancy, baby’s year of birth, nipple anatomy problems, and previous breast-feeding. Interaction was evaluated with the likelihood ratio test. If the *p-*value was ≤ 0.10, the corresponding interaction term was considered for further analysis. Previous breast-feeding had an important interaction with DDE. After stratifying on previous breast-feeding, no other interactions met the criteria.

### Reasons for weaning

Lactation may stop for a number of reasons, only some of which are relevant to our hypothesis. We asked the mother the reason she had not been breast-feeding or had interrupted breast-feeding. In a secondary analysis, participants who reported stopping for reasons unrelated to the hypothesis were censored at the time that they reported weaning; the assumption was that the pair would have continued with breast-feeding had not some external cause intervened, because there was no biological failure of the lactation. Reasons that were considered unrelated to the hypothesis included mother and child separation, use of hormonal contraception, and pregnancy. Because almost all the participants gave more than one reason, combinations that included these external events were censored also.

## Results

Participants were young (mean, 24 years of age), few attended college, and most lived in the city and its surrounding areas (Table 1). Because Tapachula is one of the poorest states in the country, the fact that 70% of the participants were in the poorest category and only 10% were not poor was expected. Children < 2,500 g of birth weight and < 36 weeks of gestational age were excluded from the original study, which explains the absence of these groups.

Those not participating (*n* = 81) were on average less exposed to DDE (median, 1.7 μg/g lipids) than were participants (median, 2.7 μg/g lipids). Among the participants, DDE was higher in women without previous breast-feeding [median, 3.36; interquartile range (IQR), 5.58] than in women with previous breast-feeding (median, 2.26; IQR, 3.29). This was expected, because DDE is eliminated via breast milk (Rogan et al. 1986).

The median duration of lactation was 10.8 months (quartiles, 5 and 17.8). About 40% of women breast-fed their child within an hour and almost all within a day after birth (Table 2). One-third introduced formula within the first month. The proportion of women with inverted nipples was lower than in a previous report from a white U.S. population (3.05%) (Park et al. 1999). Few women reported problems initiating breast-feeding. Most did not work outside the home.

**Figure 1:**
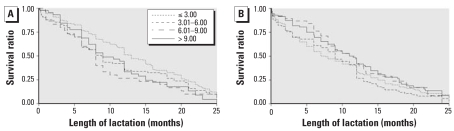
Kaplan-Meier survival curves by DDE concentration categories (μg/g lipids) in women with (*n* = 416) (*A*) and without (*n* = 368) (*B*) history of previous lactation. The graph was truncated at 25 months.

The median duration of lactation according to category of DDE was 12.0 months (≤ 3.00 μg/g), 9.0 months (3.01–6.00 μg/g), 9.0 months (6.01–9.00 μg/g), and 11.0 months (> 9.00 μg/g). Among women with no previous breast-feeding, those most exposed had the longest median duration (≤ 3.00 μg/g, 7.4 months; 3.01–6.00 μg/g, 9.0 months; 6.01–9.00 μg/g, 11.5 months; and > 9.00 μg/g, 11.3 months), whereas among women with previous breast-feeding, those least exposed had the longest median duration (≤ 3.00 μg/g, 12.6 months; 3.01–6.00 μg/g, 8.8 months; 6.01–9.00 μg/g, 8.0 months; and > 9.00 μg/g, 9.0 months) (Figure 1).

Unadjusted hazard ratios (HRs) showed similar results (Table 3); a statistically significant increase in the hazard of weaning with an increase of DDE levels was seen only in women who previously breast-fed. Adjustment for potential confounders yielded essentially the same results. Adding an interaction term between previous breast-feeding and DDE (data not shown) produced similar results; the interaction term was significant (*p* = 0.007), and the magnitude of the DDE effects was similar. We also evaluated DDT exposure, which showed no association with duration of lactation in any of the models (data not shown). Results from a secondary analysis, censoring women who weaned for reasons not considered related to DDE exposure, were similar.

We also explored whether DDE was associated with problems initiating breast-feeding. The adjusted odds ratio per unit increase in DDE levels (natural log scale) for women who had not breast-fed previously was 1.96 [95% confidence interval (CI), 1.18–3.26] and for women who had was 0.97 (95% CI, 0.60–1.57). Results for DDT were similar (data not shown).

## Discussion

Our results do not support an association between length of lactation and DDE or DDT. A statistically significant increase in the hazard of weaning associated with DDE was observed only in women who previously breast-fed, which probably reflects a non-causal mechanism. If the association between DDT and duration of breast-feeding were causal, it should also be present among women who had never breast-fed.

The present results are similar to those reported in another region (Durango) of Mexico (Gladen and Rogan 1995). In that study, an association was observed only in women who previously breast-fed. In a recent study among low-income Mexican-American mothers from an agricultural region of California, no association was seen (Weldon et al. 2006). However, our results differ from a prospective study conducted in North Carolina, which showed an association in those with no previous lactation (Rogan et al. 1987). A small study in Michigan with no contemporaneous measure of DDE also showed an association, but only in women who did not smoke during pregnancy (Karmaus et al. 2005). Using approximate conversions of milk to serum, we compared exposures in these studies. Median exposure was higher in Durango (4.2 μg/g) than in our study (2.7 μg/g), whereas U.S. women had lower medians (North Carolina, 1.7; California, 1.1; and Michigan, ~ 1.0 ) (Karmaus et al. 2005; Weldon et al. 2006). However, the proportion with high levels of DDE (> 8 μg/g lipids) was larger in our study (17%) than in the Durango study (9%), increasing our power. There is no obvious explanation for the differences between the current study and the very similar study in North Carolina. Although the previous authors used weeks predominantly breast-fed as the outcome, results using total lactation were similar (Chen and Rogan 2003). The earliest study on DDE and duration of lactation (Rogan et al. 1987) was done when levels of other organochlorines would have been higher than in the present study. Any association with DDE could have been attributed to confounding by such compounds. However, Rogan et al. (1987) measured polychlorinated biphenyls, which were not related to duration of lactation. Another potential reason for a difference between the studies was that because the study population has been exposed to DDT for generations, they may comprise a group that is no longer sensitive to DDT’s effects: Susceptible subjects may not have been able to reproduce (Longnecker et al. 2005; Venners et al. 2005). Yet another alternative explanation for the null results is that we studied very poor women, who must breast-feed because they cannot buy other food. Richer women have more options, and may be more inclined to stop breast-feeding at the first sign of problems.

If DDE affects lactation, it may affect only establishment of lactation. Milk supply is under endocrine control for the first 2–3 days; once lactation is established, it is more under autocrine (milk removal) than hormonal control (Lawrence and Lawrence 2005; Neville et al. 2001). If DDE decreased milk production in the first days, but women were nevertheless able to establish lactation, DDE may not affect duration of lactation. Estrogens act this way; they are effective for preventing milk production initially, but less effective for stopping an established lactation (Anonymous 1977). Therefore, we examined whether DDE was associated with difficulties initiating breast-feeding. We found that DDE increased the risk of difficulty initiating breast-feeding, but only in women who had not breast-fed previously. Such women may be more susceptible to endocrine disruption. Even if DDE impairs initiation, nearly all women in this study were able to breast-feed (> 98%). Among the few (*n* = 11) not able to breast-feed, the median DDE level was increased (4.8 μg/g; IQR, 5.1). Although this is consistent with the endocrine disruption hypothesis, the data are too sparse to draw any conclusions. Future studies involving women from areas where DDT is currrently used (WHO 2006) may be necessary to corroborate this preliminary result.

The results could be biased if the children who died (*n* = 8) were highly exposed, unable to breast-feed, and consequently became terminally ill. However, a serious bias is unlikely. Four of these children were in the lowest category of DDE exposure; of the remaining four, two were known to have breast-fed at least 9 months. Thus, this potential bias is unlikely to explain the observed results.

DDE levels were determined from serum drawn at delivery, using standardized methods with a low between-assay coefficient of variation. The study was double blind: Neither interviewers nor participants knew the DDT or DDE levels. Therefore, there is little reason to expect important information bias. Any errors should be nondifferential, causing bias toward the null. Length of lactation was recorded within 7 months after weaning in half of participants, and within 24 months for 96% of women. Errors should again be non-differential and biased toward the null. Moreover, reliability of long-term recall of breast-feeding duration is high (*r* = 0.86) (Tomeo et al. 1999). A limitation of the current study was that we included only male infants, because this study was derived from a previous one limited to males. However, there is no reason to suppose that any direct effect of DDE on lactation would be different in boys and girls. Perhaps more important was the exclusion of preterm and low-birth-weight infants. If initiation of breast-feeding is more difficult among such infants, this exclusion may have diminished our ability to detect any effect.

In conclusion, our relatively large study in a highly exposed area of Mexico did not support the hypothesis that exposure to DDE shortened length of lactation. The association seen in women who previously breast-fed was likely attributed to a noncausal mechanism. Nonetheless, whether DDT use has other important adverse effects on humans is still an open question.

## Figures and Tables

**Table 1:** Demographic characteristics (%) of the mother–son pairs from Tapachula, Chiapas, Mexico, 2002–2005.

Characteristic	Participants (*n* = 784)	Nonparticipants (*n* = 81)	Previous lactation (*n* = 416)	No previous lactation (*n* = 368)
Mother’s age at birth of child (years)				
15– < 20	23.0	33.3	10.1	37.5
20– < 25	35.7	37.0	35.6	35.9
25– < 30	28.1	21.0	35.8	19.3
≥ 30	13.3	8.6	18.5	7.3
Mother’s education (years)				
None	4.2	6.2	6.0	2.2
1–6	30.7	46.9	35.8	25.0
7–9	30.7	25.9	32.0	29.3
10–12	24.2	13.6	18.5	30.7
> 12	10.1	7.4	7.7	12.8
Mother’s BMI before pregnancy (kg/m^2^)				
≤ 18.5	7.0	4.9	6.5	7.6
18.6– < 25.0	57.9	46.9	53.8	62.5
25.0– < 30.0	25.3	16.0	28.4	21.7
≥ 30	9.8	6.2	11.3	8.2
First prenatal consultation				
1st trimester	42.2	24.7	40.4	44.3
After 1st trimester	21.4	19.8	21.6	21.2
None	36.4	55.6	38.0	34.5
Type of delivery				
Vaginal	62.4	66.7	63.2	61.4
Cesarean	37.6	33.3	36.8	38.6
Primiparous				
Yes	41.3	46.9	—	88.0
No	58.7	53.1	100.0	12.0
Mother’s smoking				
Ever	20.3	14.8	17.1	23.9
Never	79.7	85.2	82.9	76.1
Mother’s alcohol use				
Ever	44.6	39.5	43.3	46.2
Never	55.4	60.5	56.7	53.8
Father lived with mother at time of birth^a^				
Yes	86.5	72.8	92.1	80.2
No	13.4	25.9	7.9	19.6
Hospital of recruitment^b^				
Social Security	49.1	28.4	47.4	51.1
Ministry of Health	50.9	71.6	52.6	48.9
Residence area				
Urban	59.3	58.0	60.3	58.2
Rural	40.7	42.0	39.7	41.8
Poverty index^a^,^c^				
Poorest	70.9	66.7	79.3	61.4
Less poor	18.9	11.1	13.9	24.5
Not poor	10.1	2.5	6.7	13.9
Birth weight (g)				
2,500– < 3,000	21.7	25.9	19.5	24.2
3,000– < 3,500	48.5	48.1	46.9	50.3
3,500– < 4,000	25.1	24.7	27.4	22.6
≥ 4,000	4.7	1.2	6.3	3.0
Gestational age (weeks)				
≤ 37	3.7	3.7	3.8	3.5
> 37– < 40	37.0	46.9	37.3	36.7
≥ 40	59.3	49.4	58.9	59.8
Baby’s year of birth				
2001–2002	49.0	66.7	49.8	48.1
2003	51.0	33.3	50.2	51.9

a Variables with missing data: < 2.5%.

b Recruitment of the participants into the original cross-sectional study from which the present group was drawn (Longnecker et al. 2007).

c Using Mexican national standards based on monthly per capita income: urban (poorest, ≤ 672 pesos; less poor, > 672 to ≤ 1,367 pesos; not poor, > 1,367 pesos) and rural (poorest, ≤ 495 pesos; less poor, > 495 to ≤ 946 pesos; not poor, > 946 pesos) (SEDESOL 2002).

**Table 2:** Breast-feeding characteristics (%) of the mother–son pairs from Tapachula, Chiapas, Mexico, 2002–2005.

Characteristic	All participants (*n* = 784)	Previous lactation (*n* = 416)	No previous lactation (*n* = 368)
Mother received instruction on how to breast-feed^a^			
Yes (no previous live birth)	29.2	—	62.2
No (no previous live birth)	11.7	—	25.0
Yes (previous live birth)	41.2	70.0	8.7
No (previous live birth)	16.7	28.6	3.3
Mother received support for breast-feeding^b^			
Yes	47.7	38.7	57.9
No	52.3	61.3	42.1
Mother had difficulty initiating breast-feeding			
Yes	15.1	10.3	20.4
No	84.9	89.7	79.6
Nipple anatomy			
Inverted	1.7	1.4	1.9
Flat	8.5	4.3	13.3
Neither	89.8	94.2	84.8
Episodes of breast problems that created difficulty in breast-feeding			
None	84.4	85.1	83.7
One	13.8	12.3	15.5
Two or more	1.8	2.6	0.8
Initiation of breast-feeding (hours after birth)^a^			
1	44.1	44.0	44.3
2	15.8	15.6	16.0
3	12.0	13.7	10.1
4–6	11.7	12.3	11.1
7–24	7.8	9.1	6.3
> 24	7.1	4.8	9.8
Baby’s age at introduction of other kind of milk (months)^a^			
≤ 1	32.3	24.0	41.6
> 1–6	32.9	35.1	30.4
> 6	23.3	26.2	20.1
No other milk	11.4	14.4	7.9
Mother took medication during breast-feeding			
Yes	29.1	32.9	24.7
No	70.9	67.1	75.3
Use of oral contraceptives during breast-feeding^c^			
Yes	1.9	1.4	2.4
No	98.1	98.6	97.6
Mother returned to work before weaning			
Yes	12.2	12.5	12.0
No	87.8	87.5	88.0

a Variables with missing data: < 1.5%.

b The support is not economic, but the help that the mother received to breast-feed the baby or to provide motivation for breast-feeding.

c Included only nonestrogenic preparations.

**Table 3:** Unadjusted and adjusted HRs of weaning from the stratified Cox proportional hazards model,^a^ Tapachula, Chiapas, Mexico, 2002–2005.

	All participants (*n* = 784)			Previous lactation (*n* = 416)			No previous lactation (*n* = 368)		
*p,p*′*-*DDE (μg/g lipids)	Percent	HR	95% CI	Percent	HR	95% CI	Percent	HR	95% CI
Unadjusted									
≤ 3.00	54.3	1.00	—	62.0	1.00	—	45.5	1.00	—
3.01–6.00	22.1	1.26	1.05–1.52	20.0	1.29	0.99–1.68	24.5	1.11	0.85–1.45
6.01–9.00	8.7	1.18	0.90–1.54	7.2	1.44	0.96–2.14	10.4	0.86	0.59–1.24
> 9.00	14.9	1.10	0.88–1.36	10.8	1.46	1.04–2.04	19.6	0.80	0.60–1.07
Adjusted models^b^									
≤ 3.00		1.00	—		1.00	—		1.00	—
3.01–6.00		1.27	1.04–1.55		1.40	1.06–1.87		1.14	0.86–1.52
6.01–9.00		1.23	0.92–1.63		1.91	1.24–2.93		0.90	0.61–1.31
> 9.00		1.17	0.92–1.49		1.76	1.22–2.53		0.91	0.66–1.26

a Baseline hazard was stratified by residence area and place of recruitment for all models.

b Adjusted by mother’s age at delivery, educational background, prepregnancy BMI, smoking, poverty index, and previous breast-feeding (yes, no).
